# Federal Children’s Bureau

**Published:** 1912-05

**Authors:** 


Federal Children's Bureau.—Both houses of Congress have adopted what is termed the Federal Children's Bureau bill. This is an important piece of legislation which will be far-reaching in its effects. Among other things, it will be the duty of this bureau to investigate and report upon all matters pertaining to the welfare of children and child life, and it shall specially investigate the questions of infant mortality, the birth rate, physical degeneracy, orphanage, juvenile courts, desertion, dangerous occupations, accidents and diseases of children, employment, and legislation affecting children in the several States and Territories. The chief of the bureau will publish the results of these investigations from time to time.

One of the principal arguments advanced by the opponents of the bill was that such a bureau would affect the rights of the States. The bureau will have no legislative or executive authority.

Another argument advanced against it was that such investigation would trespass upon the sacred precincts of the home. The bill especially states that no agent or representative of the bureau shall, over the objection of the head of the family, enter any house used exclusively as a family residence.

This is a beginning. Of course, it does not meet the requirements, but when Congress shall have been awakened to its senses and a realization of the fact that the children are the seeds of the next and all future generations, and that the public health is the most valuable asset of a nation, it will be improved upon. For the present, yes, let it be slurred over and put even below the pigs in importance, in a bureau, in the Department of Labor, but when we get a *Department of Public Health,* as we surely should and *will,* the children's interest and welfare will be looked after by professional men of the best ability, and not by laymen.

I am sorry to learn that one of our Texas Senators opposed the passage of the bill.

